# Detection of Movement Events of Long-Track Speed Skating Using Wearable Inertial Sensors

**DOI:** 10.3390/s21113649

**Published:** 2021-05-24

**Authors:** Yosuke Tomita, Tomoki Iizuka, Koichi Irisawa, Shigeyuki Imura

**Affiliations:** 1 Department of Physical Therapy, Graduate School of Health Care, Takasaki University of Health and Welfare, Takasaki 370-0033, Gunma, Japan; 1930201@takasaki-u.ac.jp (T.I.); irisawa@takasaki-u.ac.jp (K.I.); s-imura@takasaki-u.ac.jp (S.I.); 2 Department of Rehabilitation, Kurosawa Hospital, Takasaki 370-1203, Gunma, Japan

**Keywords:** inertial measurement unit, movement analysis, long-track speed skating, validity

## Abstract

Inertial measurement units (IMUs) have been used increasingly to characterize long-track speed skating. We aimed to estimate the accuracy of IMUs for use in phase identification of long-track speed skating. Twelve healthy competitive athletes on a university long-track speed skating team participated in this study. Foot pressure, acceleration and knee joint angle were recorded during a 1000-m speed skating trial using the foot pressure system and IMUs. The foot contact and foot-off timing were identified using three methods (kinetic, acceleration and integrated detection) and the stance time was also calculated. Kinetic detection was used as the gold standard measure. Repeated analysis of variance, intra-class coefficients (ICCs) and Bland-Altman plots were used to estimate the extent of agreement between the detection methods. The stance time computed using the acceleration and integrated detection methods did not differ by more than 3.6% from the gold standard measure. The ICCs ranged between 0.657 and 0.927 for the acceleration detection method and 0.700 and 0.948 for the integrated detection method. The limits of agreement were between 90.1% and 96.1% for the average stance time. Phase identification using acceleration and integrated detection methods is valid for evaluating the kinematic characteristics during long-track speed skating.

## 1. Introduction

Long-track speed skating is a skillful sport where athletes glide on a 400-m ice rink at a speed of more than 50 km/h. The athletes accelerate their body using the ground reaction force exerted by the ice through an approximately 1 mm wide blade attached to the bottom of the skate shoe. Various studies have identified kinematic features of different movement phases, such as changes in the knee and trunk angles during races [1,2], that may influence the performance of long-track speed skating athletes. Additionally, a smaller push-off angle, which is the angle of the shank with respect to the floor in the frontal plane, has been shown to be associated with a greater power output [3] and skating velocity during a 5000-m race [1,2], although such a relationship was not observed during a 1500-m race [2]. Changes in the blade tilt angle during a 4000-m long-distance skating event have also been reported [4]. Several studies have also demonstrated the benefit of a greater knee flexion angle before the push-off to generate increased kinetic energy [3,5,6].

The majority of the studies that have investigated kinematic features during long-track speed skating has primarily used video analysis [1,2,3,4,5,6]. However, conventional kinematic measurements using video analysis have several limitations [7]. First, video analysis is largely influenced by the visibility of body landmarks. However, landmark visibility is often interfered by people or objects during long-track speed skating competitions or training sessions. Therefore, researchers need to synchronize measurements with multiple cameras and/or incorporate special environmental conditions to quantify kinematics with good body landmark visibility. Consequently, most kinematic analyses of long-track speed skating only measure an isolated segment of an entire race [1,2]. Although one study showed significant variability in the knee joint angles of athletes with similar performance levels [8], the source of the variability may partly be explained by the limited precision of the measurements.

Second, video analysis requires an enormous amount of time for data processing, which includes the identification of movement phases and digitization of body landmark positions. This limits the use of acquired data for immediate feedback to the athletes. Therefore, feedback regarding skating techniques must be provided predominantly based on visual or qualitative assessments from observers, without the benefit of a quantitative assessment.

In an effort to solve these limitations, inertial measurement units (IMUs) have been used increasingly in recent years as an alternative method to measure kinematics in various sports, such as speed skating, running and skiing. An IMU utilizes three axial accelerometers, gyroscopes and geomagnetometers [7]. The validity of IMUs for gait event detection has been well established [9,10], while evidence is limited for movement phase detection during sports performance. IMUs have several advantages over conventional video analyses. First, IMUs are not restricted by the visibility of body landmarks because the IMU system does not use positional data to compute kinematic outcomes. This allows for a kinematic measurement in a crowd and for a wide range of performance areas. Second, a kinematic analysis with IMUs does not require the digitization of body landmarks, allowing for a real-time display of kinematic features, including the angular velocity, acceleration and joint and segment angles. Therefore, the IMU is a promising tool for use in kinematic data acquisition in various situations, including sporting events and clinical rehabilitation.

The validity of joint angles derived from IMUs during gait and running has been widely examined by comparing them with gold standard measurements (e.g., an optical 3D motion capture system and a magnetic motion capture system) [11,12,13]. However, the validity of IMUs for the identification of movement phase classifications during long-track speed skating remains unknown. While no standardized movement phase classifications exist for long-track speed skating, foot contact and foot-off of each leg represent the start of the stance and swing, respectively, and both are important features for characterizing skating strokes [4]. Therefore, in this study, we focused on the identification of foot contact and foot-off. The objective of this study was to estimate the accuracy of IMUs for identification of foot contact and foot-off in competitive speed skaters during long-track speed skating by comparing the method with phase identification using the foot pressure sensor system. The validation of IMUs would advance the applicability of the system for use during long-track speed skating competitions and training sessions to allow for comprehensive kinematic analyses in flexible environments and instant feedback to athletes.

## 2. Materials and Methods

### 2.1. Participants

Twelve healthy competitive athletes on a university long-track speed skating team participated in the study after signing an informed consent form. All the participants had more than 10 years of speed skating experience. The demographic characteristics of the participants are shown in Table 1. This study was approved by the ethics committee of the Takasaki University of Health and Welfare (approval number: 1904) in accordance with the Declaration of Helsinki. The participants had no musculoskeletal or neurological pathologies that could affect task performance.

### 2.2. Data Acquisition

Data recording was performed during a full-speed 1000-m skating event from a static start position on a 400-m, two-lane indoor oval (Meiji Hokkaido-Tokachi Oval, Obihiro, Hokkaido, Japan).

The kinematic data were acquired using eight IMU sensors at a sampling rate of 100 Hz (myoMOTION, Noraxon, Scottsdale, AZ, USA). A data logger was embedded in each sensor, allowing data recording over a wide area. The IMU sensors were attached with double-sided tape to the skin at standardized locations on the lower thorax and pelvis and bilaterally on the thighs, shanks and feet. The specific sensor locations are shown in Table 2. Subsequently, the subjects wore compressive racing suits designed specifically for the body shape of each of the individual participants, which ensured that displacement of the sensors did not occur while recording was taking place. The foot sensors on the skating shoes were also stabilized with tape. The sensor locations were marked on the skin or skating shoes with a pen, as each sensor was attached, and we verified that there were no changes in the sensor locations before and after the data recordings.

The kinetic data were acquired using a portable foot pressure measurement system at a sampling rate of 100 Hz (F-Scan System, TeckScan, South Boston, MA, USA). The system consists of two sensor sheets, two cuff units, one data logger unit and two cables connecting the cuff units and the data logger. Two sensor sheets were trimmed to the participant’s foot size and a sensor sheet was inserted and attached to the sole of each of the skate shoes using double-sided tape. The cuff units were stabilized at the middle shanks and the data logger was attached to the back waist using Velcro tape. The IMU and foot pressure systems were synchronized using an electrical synch signal.

### 2.3. Data Analysis

We excluded the data from the first and last straights (first and last 50 m) and the first curve (100-m) from the analysis because the skating technique during these segments differs substantially from the remaining segment. Therefore, we analyzed the data for the remaining 800-m (400-m straight and 400-m curve) segment. The data from each side (left and right) were analyzed separately for both the straight and the curve. We adopted three types of analytical methods to detect foot contact and foot-off (Table 3).

**Figure 1:**
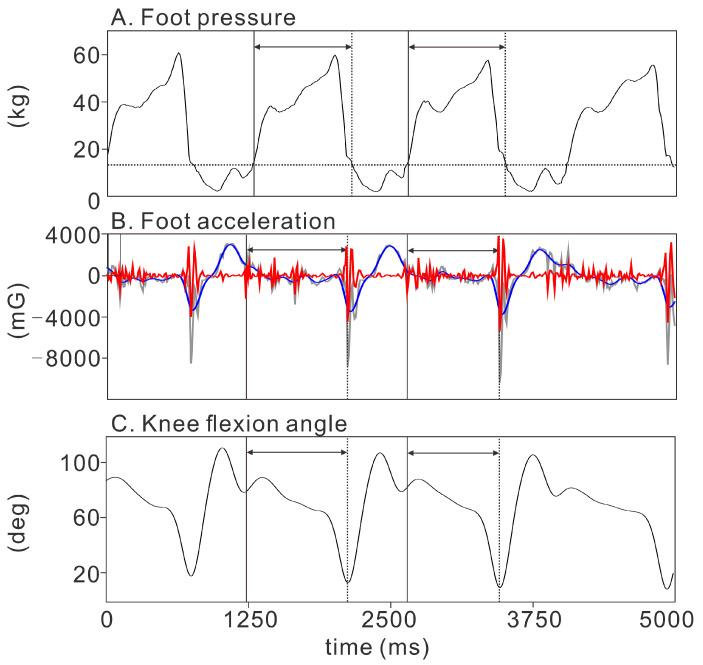
The timing identifications using three different detection methods. The vertical solid and dotted lines in each panel show the foot contact and foot-off timing, respectively. We calculated the stance time by computing the duration of the foot contact and foot-off for each skating stroke (intervals within horizontal arrows). (**A**) **Kinetic detection using the foot pressure**. The horizontal dotted line indicates the threshold level (20% peak) for the identification of foot contact and foot-off. (**B**) **Acceleration detection using the sagittal foot acceleration**. Gray line: raw sagittal acceleration. Red line: high-pass filtered sagittal acceleration. Blue line: low-pass filtered sagittal acceleration. (**C**) **Integrated detection using both the foot sagittal acceleration and the knee flexion angle**.

The first detection (the kinetic detection method) was based on the foot pressure data. Foot contact and foot-off were defined as the moments in which the foot pressure exceeded (foot contact; the vertical solid line in Figure 1A) and diminished below (foot-off; the vertical dotted line in Figure 1A) 20% of the peak foot pressure, which was calculated from all evaluated strokes (the horizontal dashed line in Figure 1A). Based on our empirical observations of the data obtained from all the participants in this study, 20% peak foot pressure was high enough to avoid false detections due to noise, but low enough to detect both the foot contact and foot-off.

The second detection (the acceleration detection method) was based on the foot sagittal (anterior-posterior direction of the foot) acceleration data obtained from the IMU sensors on each foot. The sagittal acceleration signal was used because it showed consistent changes at both the foot contact and foot-off throughout the entire 1000-m of skating. The measured sagittal acceleration signals were filtered and decomposed to their high-frequency (Butterworth high-pass filter at a cut-off frequency of 20 Hz; the red line in Figure 1B) and low-frequency (Butterworth low-pass filter at a cut-off frequency of 10 Hz; the blue line in Figure 1B) components. The high-frequency component represents instant acceleration changes and clearly shows foot contact (the vertical solid line in Figure 1B) and foot-off (the vertical dotted line in Figure 1B). The low-frequency component represents slower acceleration changes and shows the swing movement of the leg. We divided the data such that each segment comprised the start and end of the swing movement based on the low-frequency component of the acceleration. We then looked for the moment at which the high-frequency component of the foot sagittal acceleration reached its peak. The first and second peaks were set as the foot contact and foot-off, respectively.

The third detection (the integrated detection method) was based on the combination of the foot acceleration and the knee flexion angle. Raw data were automatically filtered using a robust fusion algorithm (Kalman filter) optimized for IMU data by the IMU software (myoRESEARCH 3.10, Noraxon, Scottsdale, AZ, USA). Four element quaternion values were derived by combining the elemental sensor component axes to estimate the angular offset of each sensor from the calibrated position in the global coordinate [14,15]. The knee flexion angle was automatically calculated using the biomechanical model adopted by the IMU system software. The bias (normalized root mean square) of the knee flexion angle, derived by the present IMU software from the angle based on the model recommended by the International Society of Biomechanics [16], has been reported to be 16.9 ± 5.1% during gait. The knee flexion angle showed a phasic pattern within each stroke (Figure 1C), allowing for clear segmentation of the strokes. We divided the data such that each segment comprised the start and end of the phasic pattern, which constituted one stroke and swing of each leg. We then looked for the moment at which the high-frequency component of the foot sagittal acceleration reached its peak. The first and second peaks were set as the foot contact (the vertical solid line in Figure 1C) and foot-off (the vertical dotted line in Figure 1C), respectively. All the timing detections were performed by combining the automated and visual identifications.

Based on previous studies that reported the validity of IMU systems to detect gait events [17,18], we calculated the stance time for each stroke (calculated as the time from foot contact to foot-off for each stroke) separately for each leg (right and left), section (straight and curve) and detection method (kinetic, acceleration and integrated detection). The data analysis was performed using custom-made programs (MATLAB 2014a, MathWorks, Natick, MA, USA).

### 2.4. Statistical Analysis

The stance time as calculated based on the kinetic detection method was considered as the gold standard measure in our study. The difference in the stance times among the three detection methods was examined by a repeated measures analysis of variance (ANOVA). The Tukey honestly significant difference test was performed for the post-hoc pairwise comparisons. We used the intra-class coefficient (ICC) to examine the similarity between the kinetic detection and acceleration/integrated detection methods by computing the ICC (2,1) and their 95% confidence intervals (95% CIs). To assess the validity of the proposed detection method, Bland-Altman plots and limits of agreement were calculated for both the acceleration and integrated detection methods, where we estimated the level of agreement between the proposed methods and the gold standard measure (i.e., the kinetic detection method). The bias between the proposed methods and the gold standard measure was calculated as the mean difference between the measurements from each method. The upper and lower limits of agreement, which defined the margin in which 95% of the differences between the methods were expected to lie, were calculated as a bias of ±1.96 SD. The precision of the limits of agreement is reported as the 95% confidence interval. SPSS ver. 21 (IBM, Armonk, NY, USA) was used for the statistical analyses. A statistical significance level of *p* < 0.05 was used for all the tests.

## 3. Results

In total, 1036 strokes (86.3 ± 10.4 strokes per participant) were analyzed in this study. The stance times detected by the three methods are summarized in Table 4. The results of the repeated measures ANOVA showed significant differences of stance time among detection methods on the right side (straight: *F* = 15.236, *p* < 0.001; curve: *F* = 92.298, *p* < 0.001). The post-hoc analysis showed that acceleration and integrated detection methods on the right side significantly overestimated the stance time by 2.4–3.6%, compared to the kinetic detection method (*p* < 0.05; Table 4). No significant difference was found between acceleration and integrated detections.

The ICC (2,1) results for each detection method are summarized in Table 5. The ICC (2,1) was ≥0.700 for the integrated detection method for all the sections on both sides, while the ICC (2,1) was 0.657 for the acceleration detection method in the curve for the left side.

**Figure 2:**
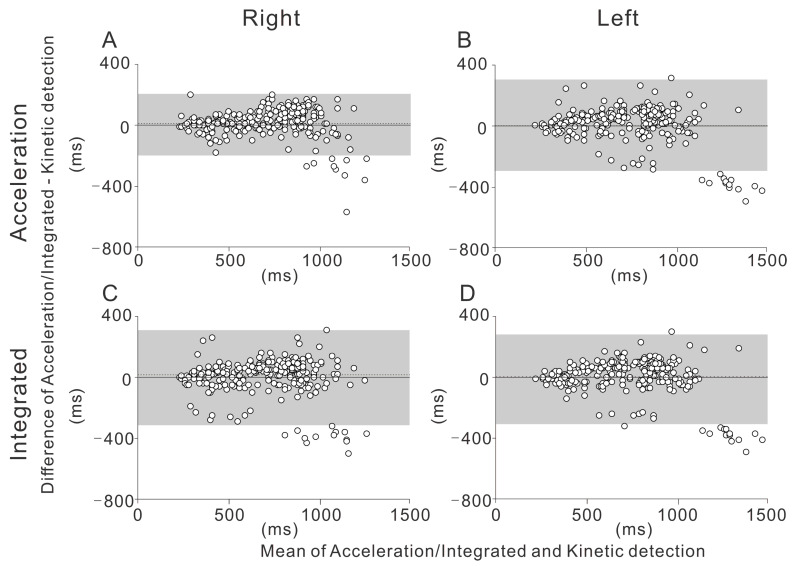
The Bland-Altman plot depicts the differences between the different detection methods in the straight, with 95% limits of agreement. The mean difference is shown by the dotted line. The 95% confidence intervals of the limits of agreement are also depicted (gray-shaded area). (**A**) Acceleration detection on the right side. (**B**) Acceleration detection on the left side. (**C**) Integrated detection on the right side. (**D**) Integrated detection on the left side.

**Figure 3:**
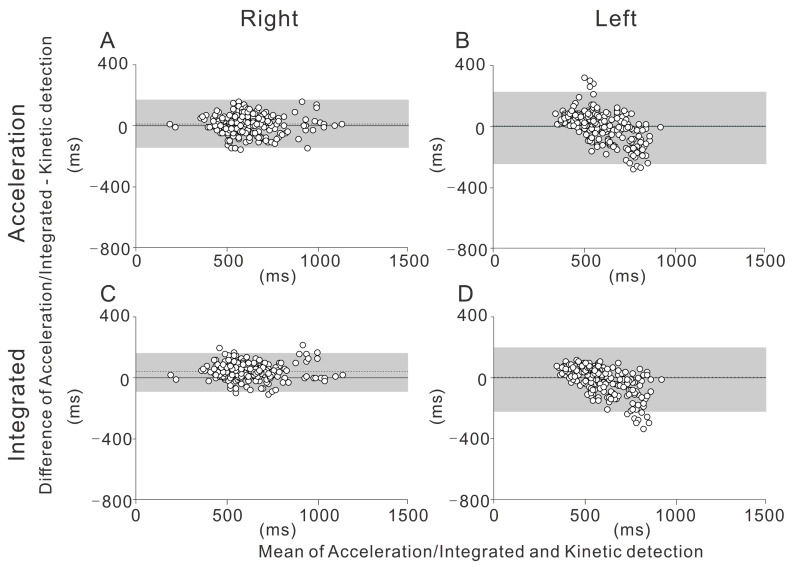
The Bland-Altman plot depicts the differences between the different detection methods in the curve, with 95% limits of agreement. The mean difference is shown by the dotted line. The 95% confidence intervals of the limits of agreement are also depicted (gray-shaded area). (**A**) Acceleration detection on the right side. (**B**) Acceleration detection on the left side. (**C**) Integrated detection on the right side. (**D**) Integrated detection on the left side.

The Bland-Altman plot is shown separately for each section (straight and curve), side (right and left), and detection method (acceleration and integrated) (Figure 2 and Figure 3).

The gray-shaded areas in the figures show the limits of agreement of the two detection methods. The proportion of cases within the limits of agreement was greater than 90% for all the measurements (LOA% in Table 4).

## 4. Discussion

In this study, we aimed to estimate the accuracy of IMUs for the phase identification of long-track speed skating for competitive speed skaters by comparing it with phase identification using the foot pressure sensor system. We examined the agreements of the acceleration and integrated detection methods with the gold standard measurements (i.e., the kinetic detection method) to calculate the stance time based on the foot contact and foot-off identified by each detection method.

The main finding of this study is the high degree of agreement between the kinetic and acceleration/integrated detection methods measured with the foot pressure sensor and IMU systems, as shown by the moderate to high ICCs. This was true for both sides (left and right) and segments (the straight and the curve). While statistically significant differences between the methods were found for the stroke time on the right side for both the straight and the curve, these differences were within 3.6%. The significant difference may partly be due to the large number of strokes used for the comparison, while the magnitude of the observed errors may not be very meaningful. Our Bland-Altman analysis shows that in the straight, the extent of the bias was proportional to the observed stance time (Figure 2). It is known that, during running, the stance time is prolonged as the running speed decreases [19]. Therefore, it should be noted that both the acceleration and integrated methods may be biased when the stance time is greater and the skating speed is slower (e.g., during long-distance skating). Our results also suggest that during the curve, the ICCs for the left side were substantially lower than those for the right side. This side-specific difference may be related to the asymmetrical skating form during the curve. Further study is needed to investigate the side-specific difference of the skating form during the curve in speed skating.

The acceleration and integrated detection methods were in significant agreement with the gold standard measure for the computation of the stance time, which suggests that the timing of the foot contact and foot-off for each leg and stroke can be accurately detected by these methods. The identification of foot contact and foot-off during skating is crucial for characterizing skating performance. It has been shown that the force measured by the sensor embedded in the skate shoe is greater when the subject stands on one leg (single leg stance), while the force substantially decreases when both legs are on the ice (double leg stance) [20]. The detection methods proposed in this study can be used to characterize skating performance using only IMUs, with minimal interference to the performance of the subject. The accuracy of acceleration and integrated methods was similar in our study, suggesting either method can be used for the detection of foot contact and foot-off. However, the phases of the speed skating motion can be divided into more details than just foot contact and foot-off [21]. IMUs have the potential to be used to identify a more detailed phase classification. In particular, the knee flexion, hip flexion and hip extension angles may potentially be used for a more detailed phase classification, as these angles show phase-dependent changes [22]. Therefore, the combined use of acceleration and the joint angle profiles obtained by IMUs would be ideal for future studies.

This study had several limitations. First, we only included healthy competitive athletes from a university long-track speed skating team. Further studies are necessary to generalize the results to different populations. Second, we used a foot pressure system as the gold standard measure, although the system itself could exhibit a measurement bias. Specifically, we used 20% peak force as the threshold for the foot contact and foot-off timing for the kinetic detection method. The 20% threshold was selected based on the observation of all trials from all participants, assuring no false detection in the kinetic detection, while the threshold may not be generalizable to other datasets. Furthermore, in reality, foot contact and foot-off occurred respectively earlier and later than the timing identified by the kinetic detection. This time lag between the actual and detected events can explain the systematic bias observed between the detection methods (i.e., all the positive ∆% values in Table 4). This time lag can overestimate the bias, while providing conservative results for the objective of this study.

## 5. Conclusions

In this study, we examined the agreement among the acceleration and integrated detection methods and the gold standard measure (i.e., the kinetic detection method) to calculate the stance time based on the foot contact and foot-off identified by each detection method. Despite the statistically significant differences between the acceleration/integrated detection methods and the gold standard measure on the right side, these differences were within 3.6%. The current data show that phase identification using acceleration and integrated detection is valid for evaluating the kinematic characteristics during long-track speed skating.

## Figures and Tables

**Table 1:** The demographics of the study participants (n = 12).

Sex (Female:Male)	5:7
Height (mean ± SD), m	165.6 ± 6.12
Body weight (mean ± SD), kg	63.46 ± 5.85
Personal best time for 1000 m (mean ± SD), sec	77.41 ± 11.76

SD: standard deviation.

**Table 2:** Sensor locations.

Lower Thoracic	In line with the spinal column at L1/T12
Pelvic	Body area of the sacrum
Thigh	Frontal and distal half (where there is less muscle displacement during motion)
Shank	Front and slightly medial (along the tibia)
Foot	Upper foot, slightly below the ankle

**Table 3:** Overview of the three analytical methods used to detect foot contact and foot-off.

Name	Type of Sensor	Type of Signal
Kinetic detection	Foot pressure	Force
Acceleration detection	IMU	Foot sagittal acceleration
Integrated detection	IMU	Foot sagittal acceleration + knee flexion angle

IMU: inertial measurement unit.

**Table 4:** Stance time detected by kinetic, acceleration and integrated detection methods.

Section	Side	Kinetic Detection	Acceleration Detection			Integrated Detection			*F*	*p*
		Mean (SD), ms	Mean (SD), ms	∆%	LOA%	Mean (SD), ms	∆%	LOA%		
Straight	Right	713.1 (243.3)	730.5 (252.2) *	2.4	95.4	738.8 (259.4) *	3.6	94.2	15.236	<0.001
	Left	736.7 (261.2)	740.2 (250.1)	0.5	91.8	744.8 (264.6)	1.1	90.1	0.670	0.512
Curve	Right	614.7 (142.6)	629.4 (153.5) *	2.4	96.1	632.3 (150.2) *	2.9	93.4	92.298	<0.001
	Left	587.6 (127.1)	587.8 (108.5)	0.0	93.8	583.3 (102.2)	0.7	95.0	0.479	0.619

The differences between the acceleration and integrated detection methods and the kinetic detection methods are shown as ∆%. The proportion of cases within the limits of agreement is shown as LOA%. The *F* value and *p* value were obtained by the repeated measures analysis of variance. * Significantly different from the kinetic detection method in the post-hoc analysis.

**Table 5:** The intra-class coefficient as computed by the acceleration and integrated detection methods.

Section	Detection Method	Right	Left
		ICC (2,1) [95% CI]	ICC (2,1) [95% CI]
Straight	Acceleration	0.927 [0.906−0.943]	0.882 [0.852−0.907]
	Integrated	0.948 [0.925−0.963]	0.868 [0.834−0.895]
Curve	Acceleration	0.904 [0.875−0.926]	0.657 [0.582−0.721]
	Integrated	0.891 [0.529−0.956]	0.700 [0.633−0.757]

ICC: intra-class coefficient; 95% CI: 95% confidence interval.

## Data Availability

Data available on request due to ethical restrictions.
